# Natural variation in codon bias and mRNA folding strength interact synergistically to modify protein expression in *Saccharomyces cerevisiae*

**DOI:** 10.1093/genetics/iyad113

**Published:** 2023-06-13

**Authors:** Anastacia N Wienecke, Margaret L Barry, Daniel A Pollard

**Affiliations:** Biology Department, Western Washington University, Bellingham, WA 98225, USA; Department of Biology, University of North Carolina at Chapel Hill, Chapel Hill, NC 27599, USA; Curriculum in Bioinformatics and Computational Biology, University of North Carolina at Chapel Hill, Chapel Hill, NC 27599, USA; Biology Department, Western Washington University, Bellingham, WA 98225, USA; Biology Department, Western Washington University, Bellingham, WA 98225, USA

**Keywords:** codon bias, mF, protein expression, interaction

## Abstract

Codon bias and mRNA folding strength (mF) are hypothesized molecular mechanisms by which polymorphisms in genes modify protein expression. Natural patterns of codon bias and mF across genes as well as effects of altering codon bias and mF suggest that the influence of these 2 mechanisms may vary depending on the specific location of polymorphisms within a transcript. Despite the central role codon bias and mF may play in natural trait variation within populations, systematic studies of how polymorphic codon bias and mF relate to protein expression variation are lacking. To address this need, we analyzed genomic, transcriptomic, and proteomic data for 22 *Saccharomyces cerevisiae* isolates, estimated protein accumulation for each allele of 1,620 genes as the log of protein molecules per RNA molecule (logPPR), and built linear mixed-effects models associating allelic variation in codon bias and mF with allelic variation in logPPR. We found that codon bias and mF interact synergistically in a positive association with logPPR, and this interaction explains almost all the effects of codon bias and mF. We examined how the locations of polymorphisms within transcripts influence their effects and found that codon bias primarily acts through polymorphisms in domain-encoding and 3′ coding sequences, while mF acts most significantly through coding sequences with weaker effects from untranslated regions. Our results present the most comprehensive characterization to date of how polymorphisms in transcripts influence protein expression.

## Introduction

Decades of research efforts have established that heritable variation in protein expression is a major driver of higher-order trait variation (Stern and Orgogozo 2008; Skelly *et al*. 2009; Chan *et al*. 2010). Advances in nucleic acid quantification technologies have facilitated numerous studies probing the effects of molecular polymorphisms on mRNA abundance variation (Brem *et al*. 2002; Rockman and Kruglyak 2006; Pai *et al*. 2012). This work established that the genetic architecture of gene expression is divided into 2 parts: a modest number of polymorphisms act in *trans* on the expression of many genes, and a large number act allele-specifically in *cis*. More recent studies have focused on protein abundances rather than mRNA abundances and found that genetic variation commonly acts specifically at the protein level, modifying either protein synthesis or decay rates (Gygi *et al*. 1999; Foss *et al*. 2011; Straub 2011; Torabi and Kruglyak 2011; Albert *et al*. 2014; Parts *et al*. 2014; Pollard *et al*. 2016). Despite enormous progress establishing that polymorphisms act in both *cis* and *trans* as well as at the mRNA level and protein level, the diversity of molecular mechanisms by which polymorphisms act on protein expression abundances remains poorly resolved (Courtier-Orgogozo *et al*. 2020; Nieuwkoop *et al*. 2020).

Codon bias, which is the uneven use of specific synonymous codons, and mRNA folding strength (mF) are 2 properties of genes that are understood to influence protein synthesis and are plausible mechanisms by which polymorphisms could act in *cis* on protein abundances (Tuller *et al*. 2011; Hanson and Coller 2018). Codon bias is generally understood to be positively associated with protein abundance because high-bias codons tend to have high abundance cognate tRNAs that result in high translational efficiency and accuracy (Ikemura 1982; Sharp *et al.* 1986; Dana and Tuller 2014; Tunney *et al*. 2018; LaBella *et al*. 2019). mF is expected to be negatively associated with protein abundance because RNA structure causes ribosome pausing (Wen *et al*. 2008; Burkhardt *et al*. 2017). However, positive associations have been observed between mF and protein abundances when comparing genes within eukaryotic genomes (Tuller *et al*. 2011; Zur and Tuller 2012). How mF might positively affect protein abundance is not well understood, but some evidence suggests that it could be related to RNA homodimerization/aggregation avoidance (Zur and Tuller 2012), ribosome recycling via RNA circularization (Park *et al*. 2013; Lai *et al*. 2018), and RNA structure refolding avoidance (Mao *et al*. 2014).

These associations between protein abundance and codon bias or mF of the transcript that encodes it have been studied using various approaches, including associating either codon bias or mF with protein abundance among genes within a genome (Zur and Tuller 2012; Dana and Tuller 2014), examining natural variation in the properties (Lawrie *et al*. 2013; Park *et al*. 2013; LaBella *et al*. 2019; Peeri and Tuller 2020), engineering alleles with artificially modified codon bias or mF (Babendure *et al*. 2006; Gooch *et al*. 2008), and computationally modeling their impact on protein expression (Tuller *et al*. 2011; Mao *et al*. 2014). However, to our knowledge, no study has systematically investigated how polymorphic variation in codon bias and mF relates to variation in protein expression in a natural population.

Investigating these factors in a population context is important for several reasons. Comparisons among alleles of the same gene, instead of comparisons across genes within the genome of an individual, minimize potential confounding effects (e.g. selection for expression levels). Because standing allelic variation is typically composed of complex combinations of genetic differences, population studies can reveal effects that are distinct from those seen from traditional single perturbation mutagenesis experiments (Greenspan 2004). Furthermore, population studies have direct relevance for understanding human population variation and for the broader goal of characterizing molecular evolutionary mechanisms.

In this study, we examined how allelic variation in codon bias and in computationally predicted mF each affect protein accumulation for 1,620 genes across 22 genetically diverse *Saccharomyces cerevisiae* isolates (Skelly *et al.* 2013). *S. cerevisiae* is known to have strong translational selection, making this a particularly good species in which to study these factors (LaBella *et al*. 2019). We found strong associations between allelic variation in codon bias and protein accumulation and between allelic variation in mF and protein accumulation. Most strikingly, we found that the effects of codon bias and mF are largely the consequence of their interaction and that this interaction is more pronounced in specific regions of transcripts.

## Methods

### Data collection and processing

From the supplemental files associated with Skelly *et al*.’s (2013) manuscript, we downloaded genome sequence, mRNA abundance, and peptide abundance data for the following 22 yeast isolates: 273614N, 378604X, BC187, DBVPG1106, DBVPG1373, DBVPG6765, L_1374, NCYC361, SK1, UWOPS05_217_3, UWOPS05_227_2, UWOPS83_787_3, UWOPS87_2421, Y12, Y55, YJM975, YJM978, YJM981, YPS128, YPS406, YS2, and YS9. The data span the set of 1,636 genes across isolates.

For each gene in each isolate, we expressed protein abundance as a sum of peptide levels (Michael J. MacCoss, personal communication, July 2018); we defined the coding sequence (CDS) based on coordinates supplied by Skelly *et al*.’s (2013) supplemental general feature format (.gff) file; and we defined 5′-untranslated region (UTR) and 3′-UTR sequences based on UTR length specifications from Tuller *et al.*’s (2009) supplemental file. The whole mRNA sequence was then the concatenation of the 5′-UTR sequence, CDS, and 3′-UTR sequence.

### Measuring protein accumulation with natural log and square root transformations of protein molecules per mRNA molecule

**Fig. 1.**
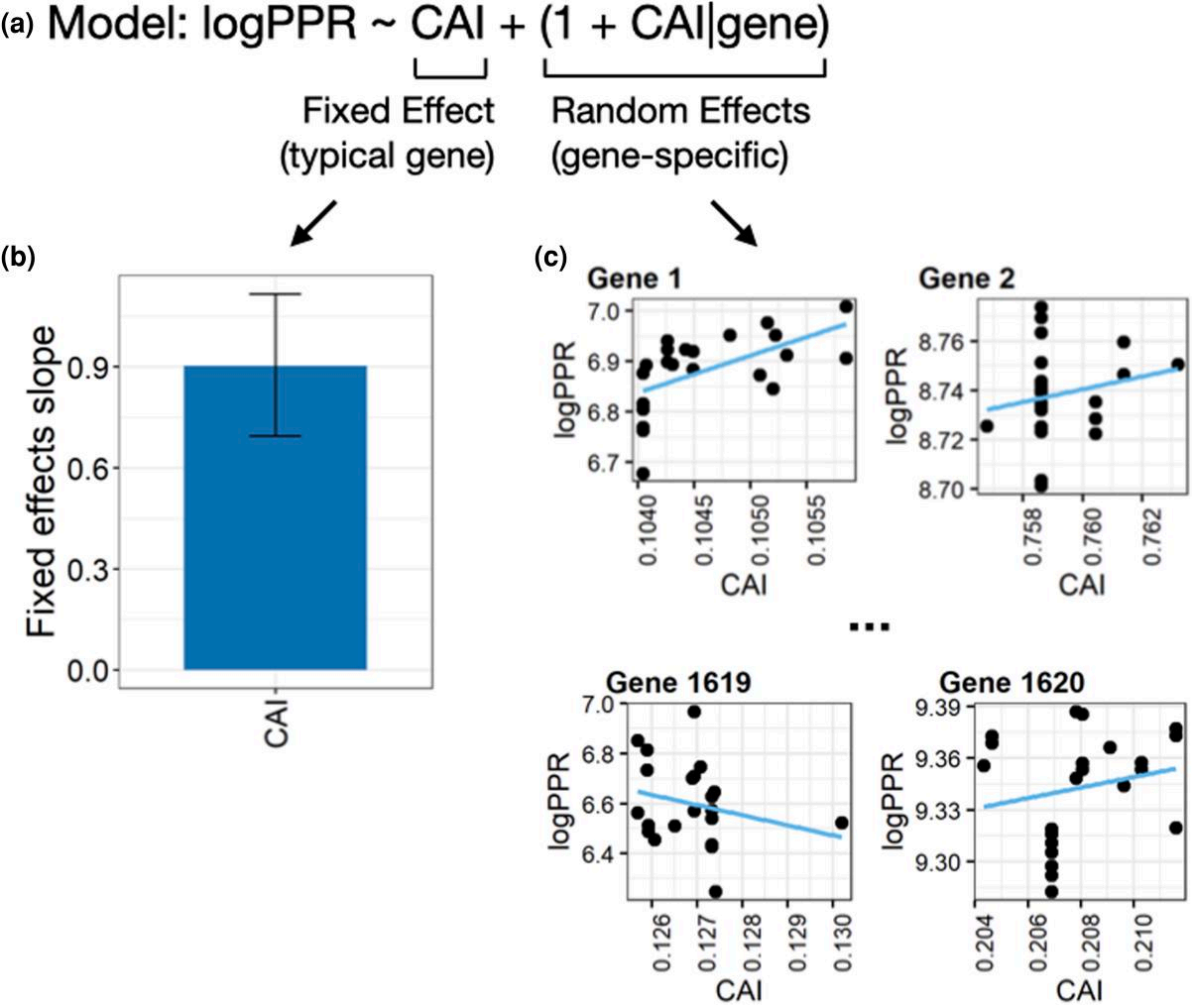
Linear mixed-effects regression model shows the association of codon adaptation index (CAI) with natural log-transformed protein molecules per mRNA molecule (logPPR) across isolates. a) Using a table of CAI and logPPR values for each of the 22 isolates for each of the 1,620 genes, the linear mixed-effects model estimates the slope and intercept for the relationship between allelic variation in CAI and logPPR across the 22 isolates. In a mixed model, there is a dependent response variable, independent fixed-effects variables that are of interest, and independent random-effects variables that contribute to the model fit but are not reported. Here, we have a dependent response variable of logPPR. We have an independent fixed effect of allelic variation in CAI on logPPR. The fixed effect represents the linear relationship between CAI and logPPR across the isolates for a typical gene. We also have independent random effects of gene-specific slopes and intercepts for the relationship of CAI and logPPR across isolates. b) Fixed-effects slope of allelic variation in CAI across 22 isolates on logPPR. The fixed-effects slope represents the association between CAI and logPPR across the isolates for a typical gene in the dataset of 1,620 genes. Error bars are the 95% confidence interval for the slope. Because the confidence interval does not include zero, the positive fixed effect of CAI is significant. c) Examples of the gene-specific relationships between CAI and logPPR for the first 2 and last 2 genes in our dataset of 1,620 genes, where points are the CAI and logPPR values for each of the 22 isolates and blue lines are the regression lines describing the linear relationship. Gene-specific slopes are used by the model to calculate the fixed-effects slope.

**Fig. 2.**
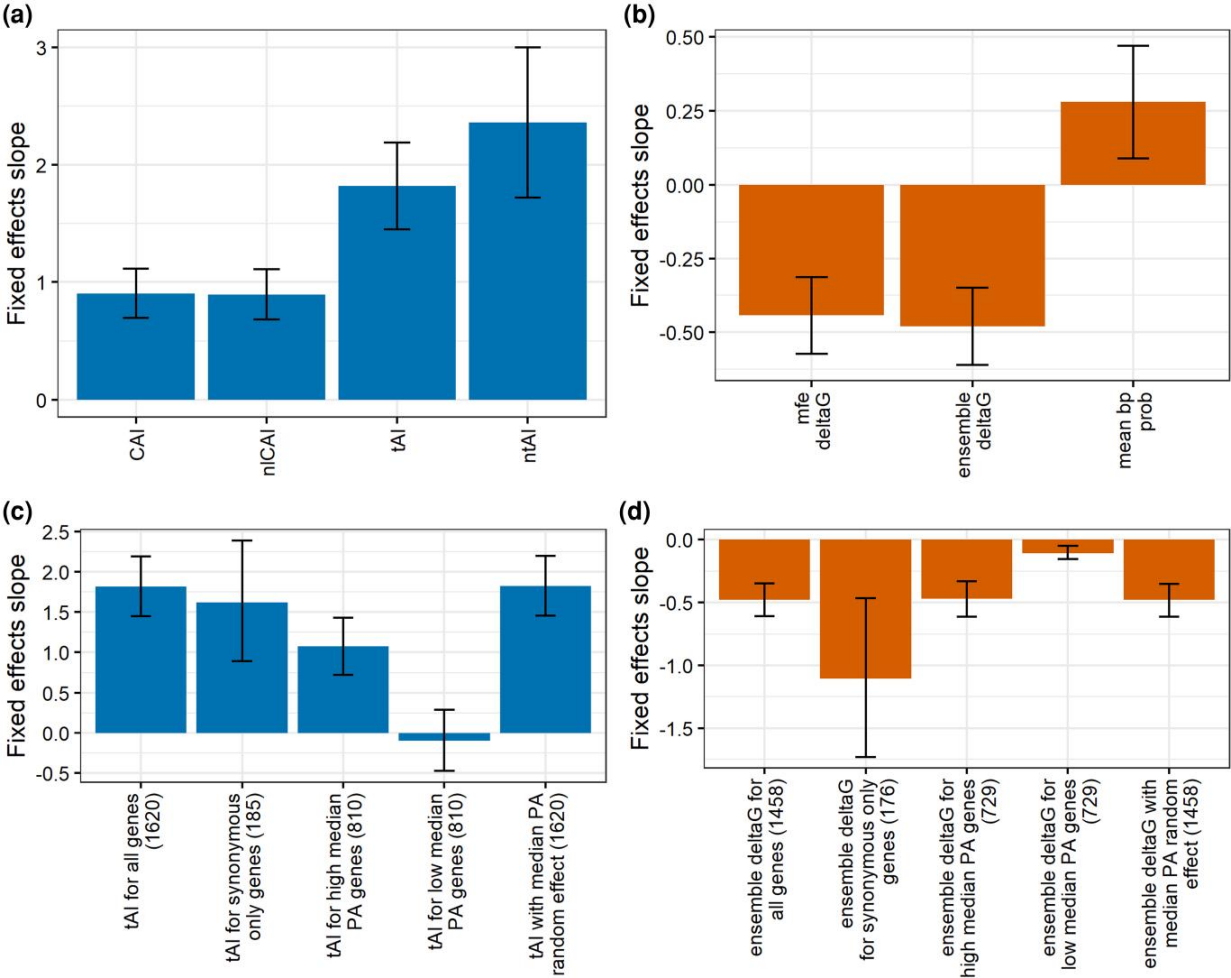
Polymorphic codon bias and mRNA folding strength (mF) are each robustly associated with logPPR. a) Fixed-effects slope of each codon bias measure (CAI, normalized-by-length CAI (nlCAI), tRNA adaptation index (tAI), and normalized tRNA adaptation index (ntAI)) is shown as the predictor of logPPR in a linear mixed-effects regression model. Models were computed using 1,620 genes, where each gene has a random-effects slope and intercept. b) Fixed-effects slope of each mF measure (mfe Δ*G*, ensemble Δ*G*, and mean base-pair probability) as the predictor of logPPR. Models were computed using 1,458 genes, where each gene has a random-effects slope and intercept. c) Fixed-effects slope of tAI as the predictor of logPPR, where each gene has a random-effects slope and intercept. Models were run with all 1,620 genes, 185 genes with synonymous and nonsynonymous polymorphisms, 810 genes with the highest across-isolate median protein abundance, 810 genes with the lowest across-isolate median protein abundance, and for all 1,620 genes with additional random effects from median protein abundance. d) Fixed-effects slope of ensemble Δ*G* as the predictor of logPPR, where each gene has a random-effects slope and intercept. Models were run with all 1,458 genes, 176 genes with synonymous and nonsynonymous polymorphisms, 729 genes with the highest across-isolate median protein abundance, 729 genes with the lowest across-isolate median protein abundance, and for all 1,458 genes with additional random effects from median protein abundance. Error bars represent 95% confidence intervals.

**Fig. 3.**
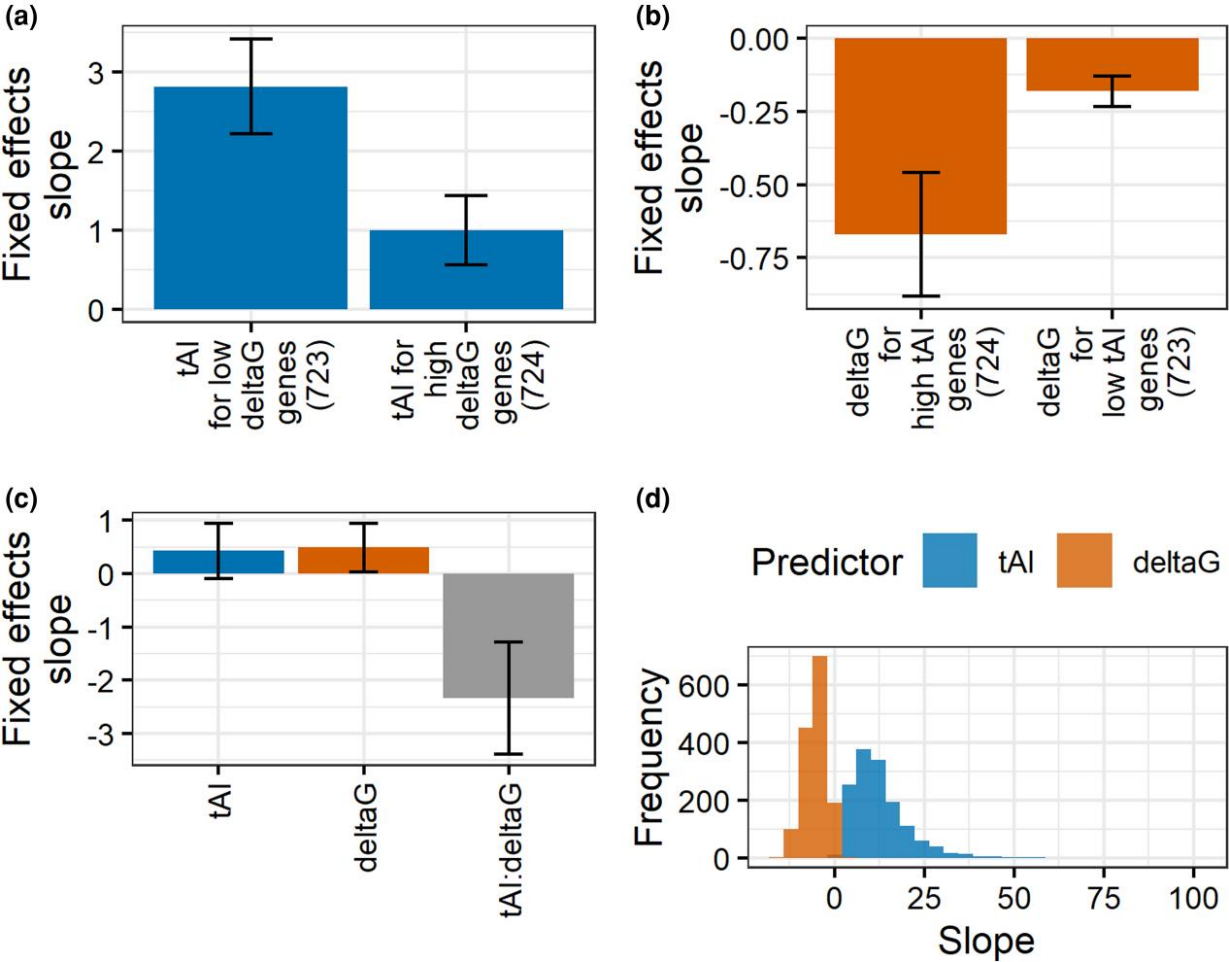
The interaction between polymorphic codon bias and mF is associated with variation in logPPR. a) Fixed-effects slope of codon bias tAI as the predictor of logPPR in a linear mixed-effects regression model for the bottom and top half of genes split by median (across alleles) mF ensemble Δ*G*. b) Fixed-effects slope of ensemble Δ*G* as the predictor of logPPR in a linear mixed-effects regression model for the bottom and top half of genes split by median (across alleles) tAI. c) Fixed-effects slope of tAI, ensemble Δ*G*, and tAI:ensemble Δ*G* interaction as predictors of logPPR in a linear mixed-effects regression model. d) Distribution across genes of the partial derivative of logPPR with respect to tAI and ensemble Δ*G* from the model with tAI, ensemble Δ*G*, and tAI:ensemble Δ*G* interaction as predictors of logPPR. Error bars represent 95% confidence intervals.

**Fig. 4.**
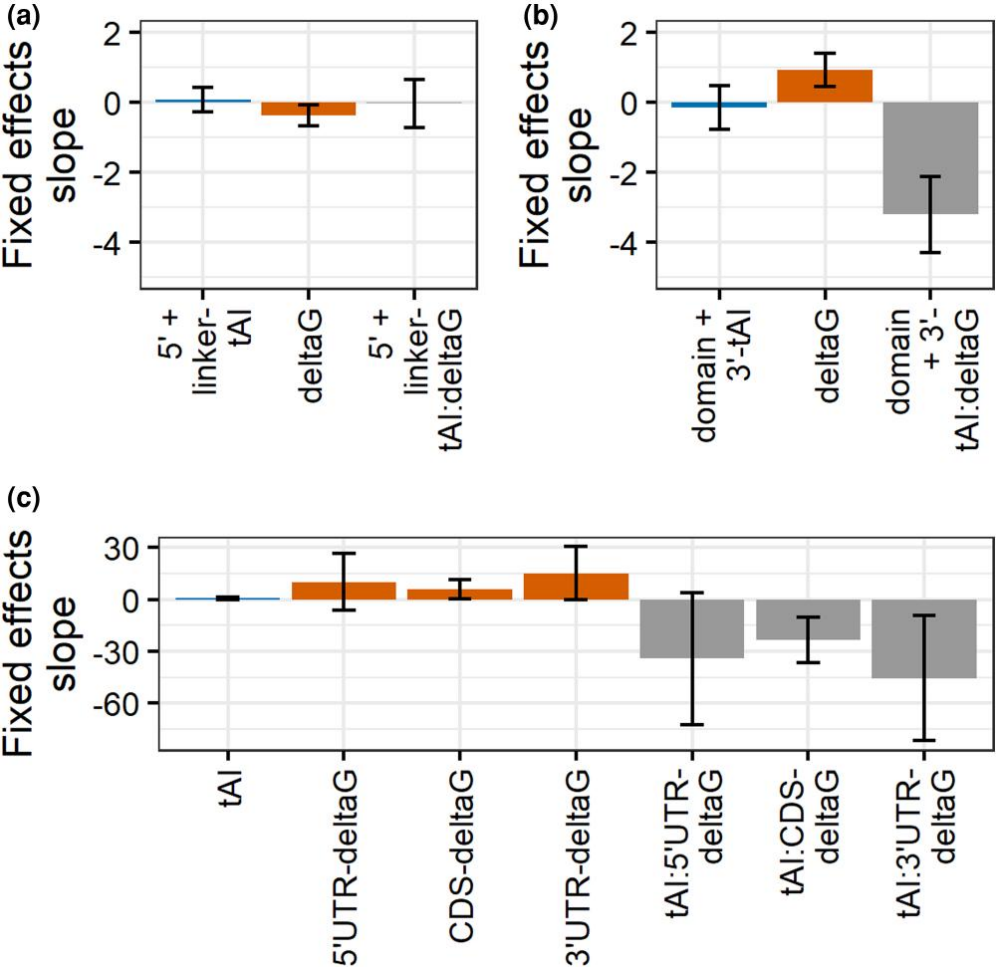
The effects of codon bias are largely due to polymorphisms localized to domain-encoding and 3′ coding regions, while the effects of mF are strongest in the coding sequence (CDS). a) To determine the localized effects of codon bias, we split CDS up into 2 regions: 5′ coding (start codon up to first domain) plus linker (sequences between domains) and domains plus 3′ coding (past last domain to stop codon). Fixed-effects slope of 5′ coding plus linker region codon bias tAI, whole transcript mF ensemble Δ*G*, and their interaction as predictors of logPPR in a linear mixed-effects regression model. b) Fixed-effects slope of domains plus 3′ coding region tAI, whole transcript ensemble Δ*G*, and domains plus 3′ coding region tAI:whole transcript ensemble Δ*G* interaction as predictors of logPPR in a linear mixed-effects regression model. c) To determine the localized effects of mF, we calculated proportional sum mfe (psmfe) Δ*G* values for substructures spanning the 5′-untranslated region (UTR), CDS, and 3′-UTR. Fixed-effects slope of CDS tAI, 5′-UTR, CDS, and 3′-UTR mF psmfe Δ*G*, and CDS tAI:5′-UTR, CDS, and 3′-UTR psmfe Δ*G* as predictors of logPPR in a linear mixed-effects regression model. Error bars represent 95% confidence intervals.

Gene-by-gene in every isolate, we measured protein accumulation as the steady-state ratio of protein abundance to mRNA abundance. Before we calculated this ratio, for each isolate, we normalized mRNA abundance and protein abundance measurements by estimates of actual cell-wide mRNA and protein molecule counts (Miura *et al*. 2008; von der Haar 2008). To do this, each gene's abundance was divided by the sum of gene abundance values for an isolate. This proportion was then multiplied by the total molecules per cell (44,155 mRNA molecules per cell; 53 million protein molecules per cell). After this normalization step, rather than PPR being in arbitrary units, the ratio is approximately in units of protein molecules per mRNA molecule (PPR). After computing PPR, we natural log-transformed PPR (logPPR) to generate a symmetrical distribution of values suitable for modeling. We chose to repeat our analysis with the square root transformation of PPR (sqrtPPR) because of the visible heteroskedasticity in the residuals vs fitted values plots of the models with logPPR as the response variable; larger fitted logPPR values had much smaller residuals than smaller fitted logPPR (Supplementary Fig. 1a). Through testing various alternative transformations of PPR (reciprocal, different base logs, exponents, etc.), we found the square root to be the only transformation of those tested that resulted in a residuals vs fitted plot with residuals appearing to be independent of the fitted value (homoskedasticity) (Supplementary Fig. 1b). Because logPPR is more readily interpretable, we represent protein accumulation as logPPR in our models (Figs. 1–4). To check the results, we also run these models with sqrtPPR as the response variable (Supplementary Figs. 2–5).

### Approximating codon bias with the codon adaptation index, normalized-by-length codon adaptation index, tRNA adaptation index, and normalized tRNA adaptation index

Three classic methods of estimating codon bias are the codon adaptation index (CAI), the tRNA adaptation index (tAI), and the normalized tRNA adaptation index (ntAI). Each relies on its own respective codon table, where every codon maps to 1 value in the range (0, 1]. A gene's CAI, tAI, or ntAI equals the geometric mean of values assigned to its comprising codons by the requisite table.

CAI quantifies a gene's tendency to use the synonymous codons most favored by a predefined training set of genes (Sharp and Li 1987). A CAI value of 1 indicates total usage of these codons, while a CAI value approaching 0 indicates complete avoidance. One approach to selecting a training set of genes is to select an arbitrary number of highly expressed genes; because of their high expression, these genes are presumed to reflect the strongest codon bias in the genome (Sharp *et al*. 1988). Ranking all genes with mRNA abundance data by their median transcript abundance (across isolates), we systematically investigated how codon usage changes as a function of selecting the 2*^i^* highest expressed genes (where *i* ∈ [1, 12]). The 3 sets with the largest number (1,024–4,096) of genes showed high frequencies of A/T-rich codons, consistent with the 2-fold mutational bias for A/T nucleotides over G/C nucleotides in *S. cerevisiae* (Lynch *et al*. 2008). The 9 sets with the smallest number (2–512) of genes showed usage of codons consistent with tRNA supplies for all amino acids except cysteine and glycine. A systematic approach to choosing a training set involves algorithmically identifying the dominant codon usage bias in the genome, independent of any expression information (Sharp *et al*. 1988; Carbone *et al*. 2003). The training set of 61 genes identified by the Carbone *et al*. (2003) algorithm for *S. cerevisiae* has codon usage similar to the most highly expressed genes and is consistent with tRNA supplies. We used this training set to calculate 1 CAI codon table per isolate. We then computed a single median CAI codon table across isolates. This is the table we use to measure the CAI of the CDS of each gene.

Normalized-by-length CAI (nlCAI) is our slightly modified version of CAI. Longer training set genes contribute more to the CAI codon table, and because all genes have their own intrinsic biases (Quax *et al*. 2015), these large contributions may misrepresent the dominant genomic level codon bias. Instead of computing the CAI codon table based on each gene's synonymous codon counts, we computed it based on each gene's synonymous codon percent abundances. Specifically, we calculated the fraction of codons that are codon *i* in each gene and added up all such fractions across genes. This gives a 61-element array, where each value matches a sense codon. For each group of synonymous codons, we divided their corresponding array values by the maximum array value within that group. In this way, we computed a single nlCAI codon table for each isolate and then take their median table for nlCAI calculations.

tAI estimates how often a gene uses synonymous codons with high supplies of cognate/near-cognate tRNAs (dos Reis *et al*. 2004). A gene always using such codons has a tAI near 1, and a gene never using such codons has a tAI near 0. This measure accounts for cases in which 1 tRNA recognizes more than 1 codon (wobble) (Crick 1966), and it approximates tRNA supply by tRNA gene copy number in the genome (dos Reis *et al*. 2004). The high positive correlation (*r* = 0.76) between the tRNA gene copy number and the tRNA abundance (in yeast) suggests that this is a reasonable approximation for our study (Tuller *et al*. 2010). Based on the approach by dos Reis *et al*. (2004), we compute a single tAI codon table and use it for tAI calculations in all isolates.

ntAI considers both the abundance of tRNAs (as measured by the tRNA gene copy number) and the abundance of codons competing for them (as measured by the sum of codon translation frequencies across all mRNAs) (Pechmann and Frydman 2013). From this view, a codon optimal for fast translation is 1 whose tRNA species are high in abundance and low in demand. A gene always using such synonymous codons has a ntAI value near 1, while a gene never using such values has a ntAI value near 0. We use the Pechmann and Frydman (2013) approach to calculate an individual ntAI codon table per isolate. For each isolate, we compute ntAI with the isolate's corresponding table.

Each measure was computed with Python (version 3.7.1).

### Approximating local codon bias with tAI

We downloaded domain coordinates, as predicted by Pfam, from the *Saccharomyces* Genome Database (SGD) (date of access: February 2019). For each gene in each isolate, we concatenated the sequences encoding Pfam-defined protein domains with the 3′ coding region (i.e. the region downstream of the 3′-most domain-encoding sequence and upstream of the translation stop codon). This is the “domain + 3′ coding” mRNA region. For the “linker + 5′ coding” mRNA region, we concatenated the sequences encoding any interdomain linkers with the 5′ CDS (i.e. the region downstream of the start codon and upstream of the 5′-most domain-encoding sequence). Using Python (version 3.7.1), we then computed tAI, our chosen measure of codon bias, for domain + 3′ coding and linker + 5′ coding regions.

### Approximating mF with mean base-pair probability, minimum free energy Δ*G*, and ensemble Δ*G*

Three gauges of mRNA folding are mean base-pair probability, minimum free energy (mfe) Δ*G*, and thermodynamic ensemble Δ*G*. All are predicted for entire mRNA transcripts (at 30°C) with the RNAfold algorithm (version 2.4.14) from the ViennaRNA Package (Lorenz *et al*. 2011).

Mean base-pair probability is the arithmetic mean of nucleotide pairing probabilities. One such pairing probability represents the chance that a given nucleotide is in a base-pair configuration, given the weighted set of thermodynamic ensemble configurations. It is calculated via the partition function (McCaskill 1990). A mean base-pair probability near 1 suggests that an mRNA's folded form is highly structured and stable.

mfe Δ*G* represents the change in Gibbs free energy an mRNA experiences after folding into its most energetically stable (mfe) configuration, as predicted by RNAfold. A negative Δ*G* value of large magnitude indicates spontaneous formation of a highly stable structure.

Ensemble Δ*G* is a Boltzmann-weighted sum of Δ*G* values: 1 Δ*G* value per mRNA structure in the mRNA's thermodynamic ensemble. Because mfe structure is only a best-guess prediction and because mRNA folding is far from static (Crothers *et al*. 1974), ensemble Δ*G* is expected to be a more accurate measure of overall mF.

### Proportional sum mean free energy Δ*G*

This method of calculation was chosen to assign Δ*G* values to regions within transcripts because it allowed us to maintain the context of the role said region has in the structure of the whole transcript. The full transcript context is critical for this study, where we are looking at the effects of polymorphisms throughout the transcript, including long-range effects. We are not aware of methods for calculating a region's Δ*G* value based on the context of the entire transcript, so we developed the following approach. To calculate mF for regions within a transcript, we used the RNAeval tool from the ViennaRNA Package (version 2.4.14) (Lorenz *et al*. 2011) to obtain a detailed thermodynamic description of each gene's mfe structure at 30°C. The algorithm reports a Δ*G* approximation for all individual substructures that make up an mRNA's overall folding shape: multi loops, external loops, interior loops, and hairpin loops. For a specific mRNA region that we wanted to compute the Δ*G* of (e.g. CDS), we summed the Δ*G* values of all the substructures, as given by RNAeval, whose entire sequence was contained in the region of interest (e.g. the Δ*G* of a hairpin loop that is made entirely within the CDS). For any substructure that contained a portion of the sequence within the region of interest (e.g. a hairpin loop involving both the CDS and the 3′-UTR sequence), we calculated what fraction of the nucleotides in the substructure belonged to our region of interest, then multiplied this value by the Δ*G* of the substructure and added this value to our existing sum. We called this value the proportional sum mean free energy (psmfe) Δ*G*.

### Gene criteria

We computed our models with different sets of genes due to limitations in the availability of data and limitations in which genes contained variation across isolates for the explanatory variables in our models. Here, we explain how these gene sets were selected.

- Of the 1,636 genes with mRNA and protein abundance data across isolates, 1,620 show 1 or more SNPs across isolates. Of these, 185 show only synonymous SNPs. To obtain the latter information, we translated the 1,636 CDS from each isolate via the translate tool from the SeqIO Biopython package (Cock *et al*. 2009). For each gene, we then aligned the corresponding set of amino acid sequences (1 sequence from each isolate) via the MUltiple Sequence Comparison by Log-Expectation (MUSCLE) algorithm (Edgar 2004). Those genes with 100% amino acid identity scores and SNP(s) across isolates were used in our 185 gene analyses. In considering model results based on this smaller set of genes, we were able to discount any effect that amino acid substitutions may have on translation rates.
- We used 1,458 of 1,620 genes in our models of global mF. These genes have available length data for the 5′-UTR and the 3′-UTR, and they have 1 or more SNPs in their concatenated 5′-UTR sequence, CDS, and 3′-UTR sequence (Supplementary Fig. 6). Of these 1,458 genes, 176 have 100% amino acid identity for our synonymous gene set.
- The intersection of the 1,620-gene set and the 1,458-gene set defines the set of 1,447 genes we used in our analyses of the synchronous actions of codon bias and mF. We ranked these 1,447 genes by their median tAI across isolates and chose the bottom 723 genes as our “low tAI” group and the top 724 genes as our “high tAI” group. This process is repeated for ensemble Δ*G* in place of tAI.
- In the models pertaining to regional codon bias, we considered a 983-gene subset of the 1,447 genes defined above. Each gene belonging to this subset is characterized by an absence of premature stop codons, by available protein domain region prediction data from Pfam, and by SNP(s) in both the domain + 3′ CDS and the linker + 5′ CDS.
- To arrive at a subset of genes suitable for regional structure models, we filtered the 1,447-gene set defined above by the following criteria to generate a 779-gene set: genes must have variation (across isolates) in local mfe Δ*G* within the 5′-UTR, the CDS, the 3′-UTR, *+1 to +10* from the 5′ cap, *−9 to +3* from translation start, *+4 to +10* of translation start, and *+1 to +18* from translation stop. Additional criteria were 5′-UTRs at least 19 nucleotides in length and 3′-UTRs at least 1 nucleotide in length.

### The linear mixed-effects regression model

We computed all linear mixed-effects regression models (Fig. 1) and log-likelihood ratio tests with the lme4 package (version 1.1.21; Bates *et al.* 2015) from R (version 3.6.0).

## Results

### Association of codon bias and protein accumulation across 22 yeast isolates for 1,620 genes

To evaluate the association of codon bias and protein accumulation, we acquired the genome sequences, transcriptomes, and proteomes of 22 genetically diverse *S. cerevisiae* isolates sampled from 6 continents and 12 types of microenvironments (e.g. bee hairs, throat sputum, fermenting palm sap, leavening bread, and forest soil) (Skelly *et al*. 2013). Transcriptome abundances and proteome abundances were measured for each haploid isolate using RNAseq and mass spectrometry in vegetatively growing cultures under phosphate limitation. We analyzed the 1,620 genes (26.22% of 6,179 total genes in *S. cerevisiae*) for which transcriptome and proteome abundances were attained in all 22 isolates.

Motivated by a paucity of knowledge about the mechanisms by which genetic variation can act in *cis* on protein synthesis and decay, we chose to examine the impact of codon bias and mF on the number of protein molecules accumulated per mRNA molecule. For each isolate's allele of each gene, we took the natural log of protein molecules per mRNA molecule (logPPR) to have a symmetric and approximately normally distributed variable to analyze in statistical models (see *Methods*). Protein expression normalized by RNA expression is often referred to as translational efficiency, and most mechanistic models connect codon bias and mF with protein synthesis rates. However, logPPR captures variation in both protein synthesis and protein stability. We therefore refrain from using the term translational efficiency and instead use protein accumulation to refer to logPPR.

We measured codon bias for each allele of each gene using the codon adaptation index (CAI). Although traditionally high-bias codons were identified using highly expressed genes (Sharp and Li 1987), we used the approach developed by Carbone *et al*. (2003) that is based on sequence information alone and is independent of gene expression data.

To understand how allelic variation in codon bias is related to allelic variation in logPPR in our dataset, we generated a linear mixed-effects regression model with logPPR as the dependent response variable, CAI as an independent fixed-effects variable, and gene as an independent random-effects variable (see Fig. 1). The linear relationship between CAI and logPPR varies among genes (Fig. 1c); however, by treating genes as a random effect in the mixed model, we can remove the gene-specific effects and evaluate how allelic variation in CAI relates to logPPR for a typical gene in the dataset (Fig. 1b). Over our dataset for 1,620 genes, we found allelic variation in CAI to have a positive association with logPPR (Fig. 1b). To evaluate the significance of this positive association, we performed a log-likelihood ratio test, which compares the fit of our model with a model lacking CAI as a fixed effect. Using this approach, we found that the positive association between CAI and logPPR was highly significant (*G* = 72.977, df = 1, *P* = 1.31e-17). From this analysis, we learned that alleles with higher codon bias tend to have higher logPPR, demonstrating for the first time that codon bias plays an important role in explaining variation in protein accumulation within natural populations.

We next examined the robustness of our result. Visualizing the residuals (difference between model prediction and actual data) with a logPPR vs residuals plot (Supplementary Fig. 1a) revealed that the residuals show some heteroskedasticity (dependence between residual values and logPPR), which is a violation of the model assumptions. In an attempt to minimize the heteroskedasticity, we applied several transformations of the ratio of protein molecules per RNA molecule, other than the natural log. We found that taking the square root of the ratio (sqrtPPR), which compresses the left tail of the distribution of PPR values, successfully minimizes the heteroskedasticity (Supplementary Fig. 1b). Acknowledging that the square root is considerably less conventional than taking the log of a ratio, we found that the association between CAI and sqrtPPR was significant (log-likelihood ratio test: *G* = 44.135, df = 1, *P* = 3.06e-11) (Supplementary Fig. 2a), suggesting that the association is not due to the violation of a model assumption. We note that we observed the same pattern of heteroskedasticity for logPPR and lack of heteroskedasticity for sqrtPPR for all models used throughout this study. We will present logPPR results while noting differences between logPPR and sqrtPPR and reporting sqrtPPR results in the supplemental figures (Supplementary Figs. 2–5).

The lengths of the 61 genes selected by Carbone *et al*. (2003) to use in the calculation of CAI vary such that some genes contribute more to the estimation of codon bias than others. To give each gene equal weight, we normalized codon frequencies across the 61 genes to calculate normalized-by-length CAI (nlCAI). The association between nlCAI and logPPR (log-likelihood ratio test: *G* = 70.982, df = 1, *P* = 3.60e-17) is negligibly different from the association between CAI and logPPR (Fig. 2a and Supplementary Fig. 2a), suggesting that length variation did not introduce substantial bias.

To further evaluate if the association between codon bias and logPPR is robust to the method used to calculate codon bias, we examined 2 additional measures of codon bias. The tRNA adaptation index (tAI) measures codon bias based on the tRNA gene copy number as an estimate of tRNA supply (dos Reis *et al*. 2004) (see *Methods*). The normalized tRNA adaptation index (ntAI) modifies tAI to also account for the demand on tRNAs by the cognate codons in the pool of mRNA (Pechmann and Frydman 2013) (see *Methods*). Thus, we used 2 measures of codon bias that are based on codon usage (CAI, nlCAI), 1 measure based on estimated tRNA availability (tAI), and 1 based on both codon usage and estimated tRNA availability (ntAI). For our full set of 1,620 genes, the associations between tAI and logPPR and ntAI and logPPR are significant and positive (log-likelihood ratio tests: tAI *G* = 95.587, df = 1, *P* = 1.42e-22; ntAI *G* = 52.268, df = 1, *P* = 4.84e-13) (Fig. 2a and Supplementary Fig. 2a). Although the slope for ntAI is the steepest, it is more reflective of the variation in the explanatory variable rather than reflective of the predictive ability of the association. Instead, we focus on the confidence interval and *P*-value for each slope as the indicator of relative strength of association. The association between tAI and logPPR was the most significant of those evaluated, suggesting that the tRNA gene copy number captures important information about the effects of codon bias on protein accumulation. For the remainder of the study, we will report on the effects of codon bias using tAI.

Most of the genes in our study have both synonymous and nonsynonymous polymorphisms. Because nonsynonymous polymorphisms are known to influence logPPR through mechanisms besides codon bias, we repeated our analysis on the 185 genes that lack nonsynonymous polymorphisms. Again, we found a significant and positive association between tAI and logPPR (log-likelihood ratio test: *G* = 18.607, df = 1, *P* = 1.61e-05) (Fig. 2c and Supplementary Fig. 2c).

Although we controlled for the effects of gene-specific properties by including gene-specific random-effects slopes and intercepts in our regression models, we next examined if the expression level of a gene influences the relationship between allelic variation in codon bias and protein accumulation. Selection favors high-bias codons in highly expressed genes (Sharp *et al.* 1986); however, we are not aware of a mechanistic reason for why polymorphic codon bias would have different effects in high vs low expression genes. To evaluate this, we split the genes in our study into 810 high expression genes and 810 low expression genes, based on median protein abundance across isolates, and built tAI vs logPPR models for each half. To our surprise, we found that the half of genes with the lowest median protein abundances have no significant association between tAI and logPPR (log-likelihood ratio test: *G* = 0.2478, df = 1, *P* = 0.619), while the half of genes with the highest median protein abundances have a significant positive association between tAI and logPPR (log-likelihood ratio test: *G* = 35.353, df = 1, *P* = 2.75e-09) (Fig. 2c and Supplementary Fig. 2c). This suggests that polymorphic codon bias impacts protein accumulation more significantly in more highly expressed genes. To explicitly control for the effect of expression levels in our model, we added median protein abundance as a random effect on the slope of tAI and logPPR (in addition to gene random effects) for the full set of 1,620 genes and found that tAI has a slightly more significant positive association with logPPR than in the model without this random effect (log-likelihood ratio test: *G* = 95.966, df = 1, *P* = 1.17e-22) (Fig. 2c and Supplementary Fig. 2c). For the remainder of the study, we included median protein abundance as a random effect on slopes in our models (Figs. 3 and 4 and Supplementary Figs. 2–5)

Overall, allelic variation in codon bias appears to be robustly associated with protein accumulation within a natural population, with the most significant effects coming from highly expressed genes.

### Association of polymorphic mF and protein per mRNA

With the relationship between codon bias and logPPR established, we next investigated the association between mF and protein accumulation across the same 22 isolates of *S. cerevisiae*. A growing body of evidence has shown the counterintuitive pattern that genes with more structured mRNAs produce more protein (see *Introduction*). For each isolate's allele of each gene in our dataset, we calculated 3 measures of mF (see *Methods*): mfe Δ*G* is an estimate of the change in Gibbs free energy an mRNA experiences after folding into its most energetically stable configuration, ensemble Δ*G* is a Boltzmann-weighted sum of estimated Δ*G* values from the most probable RNA structures, and mean base-pair probability is the mean chance that a nucleotide is base-paired, given the weighted set of ensemble configurations. We found that 1,458 genes have allelic variation for these mF measures, and we used these 1,458 genes to evaluate the association between mF and logPPR. All 3 measures of mF are significantly and positively associated with logPPR (Fig. 2b and Supplementary Fig. 2b). We note that because more negative Δ*G* represents more stable structures, we will describe a negative slope for Δ*G* vs logPPR as a positive association between mF and logPPR. Ensemble Δ*G* shows the most significant association with logPPR (log-likelihood ratio test: *G* = 51.861, df = 1, *P* = 5.96e-13); mfe Δ*G* shows nearly as significant an association (log-likelihood ratio test: *G* = 44.507, df = 1, *P* = 2.53e-11); mean base-pair probability shows a less significant association (log-likelihood ratio test: *G* = 8.2598, df = 1, *P* = 4.05e-03). For the remainder of the paper, we will report on the effects of mF as measured by ensemble Δ*G*, except where noted.

To control for potential impacts of nonsynonymous polymorphisms, we repeated our analysis on the 176 genes that have variation in mF and only synonymous polymorphisms. For this set of genes, we found that ensemble Δ*G* is significantly and positively associated with logPPR (log-likelihood ratio test: *G* = 11.204, df = 1, *P* = 8.16e-04) (Fig. 2d and Supplementary Fig. 2d).

Because previous studies have established that gene-to-gene variation in mF is correlated with protein abundance (Zur and Tuller 2012), we split the 1,458 genes into the half with the lowest median protein abundances and the half with the highest median protein abundances and ran the ensemble Δ*G* vs logPPR model on each set of 729 genes. Our understanding of the relationship of mF and protein abundance among genes was that high mF is selected for in highly expressed genes. We had no expectation that gene-to-gene variation in protein abundance would influence the association of mF and logPPR. Both sets of genes have significant associations (Fig. 2d and Supplementary Fig. 2d), with the high median protein abundance set having the more significant association (log-likelihood ratio test: high median protein abundance *G* = 40.856, df = 1, *P* = 1.638e-10; low median protein abundance *G* = 12.403, df = 1, *P* = 4.287e-04). Thus, it appears that the impact of the expression level of a gene on the relationship of mF with logPPR is more subtle than it is for codon bias but nonetheless has an effect. To control for this, we added median protein abundance as a random effect on the slope of ensemble Δ*G* vs logPPR for our full set of 1,458 genes and found that mF has a more significant association with logPPR than it does for the model lacking median protein abundance as a random effect (log-likelihood ratio test: *G* = 51.906, df = 1, *P* = 5.821e-13) (Fig. 2d and Supplementary Fig. 2d). For all mixed-effects regression models for the rest of this study, we controlled for the impact of gene-to-gene variation in expression levels by including median protein abundance as a random effect.

In summary, our results show that allelic variation in mF appears to have a robust positive association with protein accumulation within a natural population.

### logPPR is predicted by an interaction between polymorphic codon bias and mF

Having established that allelic variation in codon bias and mF each are associated with protein accumulation, we next examined if codon bias and mF operate independently or if their effects arise from an interaction. In several species, codon bias and mF are positively correlated (Peeri and Tuller 2020), suggesting shared selection pressures and possibly interacting mechanisms. A simulation study by Mao *et al*. (2014) concluded that codon bias has the biggest impact on translation rates when mF is high. They hypothesized that this interaction arises when high mF causes ribosomes to become so densely packed that the mRNA molecule does not refold between adjacent ribosomes and instead becomes linearized, leaving codon bias as the rate-limiting factor in translation rates. To test Mao *et al*.’s (2014) prediction, we analyzed the 1,447 genes polymorphic for both codon bias and mF. We used tAI to quantify codon bias and ensemble Δ*G* for mF because they were found to be the most significant predictors of logPPR (see Fig. 2a and b). We computed the overall mF of a single gene as the median ensemble Δ*G* across the alleles of the gene. We found that indeed, the top half of genes, ranked from most stable overall mF to least stable, show a much stronger relationship between polymorphic tAI and logPPR than the bottom half of genes (Fig. 3a and Supplementary Fig. 3a). Although not a stated prediction of Mao *et al*. (2014), for completeness, we examined if the reciprocal interaction was occurring. Specifically, we wanted to determine whether highly codon-biased genes show a stronger relationship between mF and logPPR. We measured the overall codon bias of each gene as the median tAI across its alleles. Interestingly, we found that the top half of genes, ranked from highest overall codon bias to lowest, show a much stronger relationship between polymorphic ensemble Δ*G* and logPPR than the bottom half of genes (Fig. 3b and Supplementary Fig. 3b). This pair of results suggests that codon bias and mF interact synergistically to influence protein accumulation.

To evaluate the interplay of individual effects and synergistic effects, we ran a linear mixed-effects model with independent terms for tAI and ensemble Δ*G* and an interaction term between tAI and ensemble Δ*G*. Consistent with codon bias and mF working synergistically, the interaction term has a significant negative slope, and including the interaction term significantly improves the fit of the model (log-likelihood test: *G* = 27.216, df = 1, *P* = 1.819e-07) (Fig. 3c and Supplementary Fig. 3c). Thus, stable mF and high codon bias together are associated with high logPPR.

It is noteworthy that the independent effects of codon bias and mF in the model with the interaction term are quite small. The tAI term is not significant, while the ensemble Δ*G* term is weakly significant and has a positive slope, consistent with mF having a weak negative effect on protein accumulation, separate from its positive effects through the interaction. The weak negative effect for mF, however, was not significant in the control model using sqrtPPR as the response variable (Supplementary Fig. 3c). Furthermore, if there is a weak negative effect from mF, it is swamped out by the positive effect from the interaction, as shown by the negative partial derivative of logPPR with respect to ensemble Δ*G* for most genes (Fig. 3d). Thus, it appears that all or nearly all of the impact of polymorphic codon bias and mF on protein accumulation is through their interaction.

With little known about the interplay between codon bias and mF, we next looked to see if the signal for the interaction is uniform across transcripts or if specific locations within transcripts are driving the relationship.

### Effects of codon bias are localized to protein domains and 3′ coding regions

Comparisons across genes have revealed that codon bias is strongest in protein-domain-encoding regions and in 3′ coding regions and weakest in 5′ coding regions and interdomain linker regions. The 3′ coding region is bordered by the 3′-most domain-encoding region and the translation stop codon; it has the highest proportion of optimal codons of all regions of the coding DNA sequence (CDS) (Tuller *et al*. 2010). Such levels of bias are thought to protect against ribosome collisions and the ensuing interruptions in protein synthesis, such as premature translation termination. It is especially costly in terms of expended energy and resources if a ribosome terminates prematurely this far past the start codon (Tuller *et al*. 2010; Plotkin and Kudla 2011). Domain-encoding regions show this pattern presumably because selection for high codon bias is unconstrained and perhaps because selection to maintain domain function additionally selects for the high-bias codons that tend to be translated more accurately (Kramer and Farabaugh 2007; Drummond and Wilke 2009; Zhou *et al*. 2009; Kramer *et al*. 2010; Geiler-Samerotte *et al*. 2011; Zhou *et al*. 2015). Although these patterns of codon bias could mean that polymorphisms that alter codon bias may have different impacts on protein accumulation depending on their location, the patterns may also be unrelated to the functional association between codon bias, mF, and logPPR.

To evaluate how location impacts the effect of polymorphisms that alter codon bias, we compared the effects of polymorphisms in 5′ coding and interdomain linker regions with those in domains and in 3′ coding regions. For each gene, we separated codons that fell into 5′ coding and linker sequences (“5′ + linker coding”) with those that fell into the domain-encoding sequence and 3′ CDS (“domain + 3′ coding”). We wanted to see how polymorphisms in these 2 subsets of codons act on their own and how they interact with mF to influence protein accumulation. Of the 1,620 genes in our dataset, 1,458 have polymorphisms that alter mF. Of those, 983 have codon-bias-altering polymorphisms in both 5′ + linker CDS and domain + 3′ CDS. For these 983 genes, we ran a linear mixed-effects regression model on logPPR vs 5′ + linker coding tAI, whole transcript Δ*G*, and the interaction of those terms (Fig. 4a and Supplementary Fig. 4a) and a separate model on logPPR vs domain + 3′ coding tAI, whole transcript Δ*G*, and the interaction of those terms (Fig. 4b and Supplementary Fig. 4b). Polymorphic codon bias in 5′ coding + interdomain linker regions has different effects on logPPR than polymorphic codon bias in domain + 3′ coding regions (Fig. 4a and b); notably, there appears to be no effect from 5′ coding + interdomain linker regions (Fig. 4a), and the effects from domain + 3′ coding regions are strong and similar to those from the whole CDS (Figs. 3c and 4b). Thus, the strong codon bias in domains and 3′ coding regions appears to be closely related to the functional association between allelic variation in logPPR, codon bias, and mF.

### Effects of mF are stronger in CDS than UTRs

Similar to codon bias, mF varies across transcript regions (Bevilacqua *et al*. 2016; Gebert *et al*. 2019) and is understood to have differential impacts on protein accumulation depending on the location of stable structures (Kozak 1986; 1989; 1990). In yeast genes, high mF is associated with increased protein yield when located *+1 to +10* bases from the 5′ cap (Kertesz *et al*. 2010; Cuperus *et al*. 2017). The mechanism for this association is not known, and the association is in contrast with observations from mammalian mRNAs (Kozak 1989; Babendure *et al*. 2006). Similarly, high mF is typically seen within the region *+4 to +10* bases from the start codon (Shabalina *et al*. 2006; Kertesz *et al*. 2010) and is hypothesized to act as a “speed bump” to improve the efficiency of start codon recognition, especially in genes with suboptimal start codon contexts (Kozak 1990). In contrast, mF tends to be quite low within *−9 to +3* bases from the start codon and in the region from the stop codon into the 3′-UTR (Shabalina *et al*. 2006; Kertesz *et al*. 2010; Wan *et al*. 2012; Peeri and Tuller 2020). Further, stable stem-loop structures located in these regions can inhibit translation (Kozak 1986; Sherman and Baim 1988; Vega Laso *et al*. 1993; Niepel *et al*. 1999; Lamping *et al*. 2013). These patterns of mF alone suggest a possible functional connection between localized mF, codon bias, and logPPR, and the studies introducing engineered alleles with locally altered mF strongly suggest a functional role.

To test if allelic differences in localized mF have different effects on logPPR, we examined the regions at the 5′ cap (*+1 to +10* bases of 5′ cap), upstream and including the start codon (*−9 to +3* bases of translation start), downstream of the start codon (*+4 to +10* bases of translation start), and downstream of the stop codon (*+1 to +18* bases of translation stop). In contrast with how polymorphisms act on codon bias, polymorphisms can act across a transcript to influence the mF of a distant region. Therefore, instead of categorizing polymorphisms based on their location, we looked to see how mF in each region changes across alleles. To do this, we needed to estimate the mF for these regions of transcripts. To our knowledge, no method exists for estimating mF for a region of a transcript based on the full transcript context, which is important for understanding the impacts of polymorphisms throughout the transcript. We therefore developed an estimate of a region's mF, called psmfe Δ*G*, which adds up the Δ*G* values of the stem-loop structures contained within a region and includes structures that partially span the region (see *Methods*). For all 4 regions, we uncovered no significant associations between logPPR and the interaction of codon bias and mF (Supplementary Fig. 5a). The lack of associations suggests that the role mF plays in these 4 specific regions of the transcript is minimal compared to the impact of mF across the entire transcript. However, the small size of these 4 regions (<18 bp) could have limited our power to detect their effects. To test this, we sampled moderately small (40 bp) regions across transcripts and found that none have significant associations (Supplementary Fig. 5b and d). Thus, within the limitations of our sample size and the accuracy of our regional mF predictions, we cannot rule out the possibility that allelic variation in RNA structures near the 5′ cap, start codon, and stop codon plays an important role in protein accumulation.

On a larger scale, the CDS of yeast mRNA is more structured than either the 5′-UTR or the 3′-UTR (Kertesz *et al*. 2010; Wan *et al*. 2012), and mF is highest at the center of the CDS (Peeri and Tuller 2020). This hallmark is both selected for (Katz and Burge 2003) and positively correlated with gene expression across genes (Zur and Tuller 2012). The high mF in CDS may boost protein expression by facilitating cotranslational protein folding (Faure *et al*. 2016) or inhibiting unproductive translation initiation within the CDS (Kertesz *et al*. 2010). Both the patterns and correlation with gene expression suggest a differential functional impact of polymorphisms in the CDS compared to the UTRs.

To test this hypothesis, we looked at the course-scale differences in mF effects between CDS, 5′-UTR, and 3′-UTR and how those local mF effects interact with whole-gene codon bias. Using the 1,312 genes with polymorphic CDS tAI and polymorphic CDS, 5′-UTR, and 3′-UTR psmfe Δ*G*, we ran a linear mixed-effects model with logPPR as a function of CDS tAI, psmfe Δ*G* for CDS, 5′-UTR, and 3′-UTR, and the interactions between tAI and each Δ*G* term. This revealed that the interaction between codon bias and mF and the independent effects of mF on logPPR are strongest in the CDS and are weaker in the UTRs (Fig. 4c and Supplementary Fig. 4c). Thus, it appears that the impact of polymorphic mF on protein accumulation is not uniform across the length of a transcript but is most significant in the CDS.

## Discussion

In this study, we investigated the association of allelic variation in codon bias and mF with allelic variation in protein accumulation in *S. cerevisiae*. We leveraged a published dataset of genome sequences, transcriptome abundances, and proteome abundances for 22 yeast isolates (Skelly *et al*. 2013), then calculated codon bias and mF from genome sequence data and measured protein accumulation as the natural log of the ratio of protein molecules per mRNA molecule (logPPR). Our choice to study logPPR was motivated by an interest in understanding how natural genetic variation can act in *cis* on protein synthesis and decay rates, an understudied topic. Evidence suggests that allelic variation in codon bias and mF could impact mRNA synthesis and decay rates (Presnyak *et al*. 2015; Bazzini *et al*. 2016; Cohen *et al*. 2018; Hia *et al*. 2019). We have assumed that if codon bias and mF cause variation in mRNA levels, this variation would carry through to protein level variation, and therefore, logPPR would not be impacted. We assert that investigating if and how allelic variation in codon bias and mF are associated with mRNA levels and if those effects are independent of the effects on logPPR would be an interesting direction for future research.

By using linear mixed-effects models, we estimated the expected slope of the response of logPPR as a function of allelic variation in codon bias and/or mF while controlling for gene-to-gene differences in levels and effects. Other studies have focused on gene-to-gene variation in how codon bias and mF impact protein expression (Sharp *et al.* 1986; Zur and Tuller 2012), and the findings of these studies motivated our analysis of isolate-to-isolate allelic variation. While variation between genes is influenced by gene-specific characteristics that have been shaped over long periods of time by evolutionary processes, our analysis of allelic variation between isolates is focused on the mechanistic effects of codon bias and mF on protein synthesis and stability. Gene-specific characteristics may modify how allelic variation in codon bias and mF is associated with protein accumulation, but they cannot be what causes the associations we have found. To control how gene-specific characteristics modify our associations, we included gene-specific differences in slopes and intercepts as random effects in our mixed models. Furthermore, we allowed the median protein expression level of each gene to modify slopes in our mixed models. Therefore, the fixed-effects results that we have reported are strictly for the impact of allelic variation in codon bias and mF on logPPR.

Previous work on codon bias and mF showed that they are each correlated with protein levels, selected for across species, and capable of altering protein levels when manipulated (Babendure *et al*. 2006; Gooch *et al*. 2008; Tuller *et al*. 2011; Zur and Tuller 2012; Park *et al*. 2013; Dana and Tuller 2014; Mao *et al*. 2014; Hanson and Coller 2018; Tunney *et al*. 2018; LaBella *et al*. 2019; Peeri and Tuller 2020). Our study shows the most comprehensive evidence to date that allelic variation in codon bias and mF in a population are both significantly associated with protein accumulation (Fig. 2 and Supplementary Fig. 2). These associations in the context of previous work motivate a deeper investigation of codon bias and mF as important *cis*-acting mechanisms of protein expression variation.

Although we controlled for differences in expression levels among the genes in our dataset, we were quite surprised to discover that median protein abundance influences how codon bias and mF are associated with protein accumulation (Fig. 2 and Supplementary Fig. 2). What we see is that polymorphic codon bias, and to some degree mF, has a bigger impact on protein accumulation in genes that are more highly expressed. Previous studies looking at gene-to-gene variation found associations of codon bias, mF, and protein abundance; for instance, genes with higher protein abundance tend to have higher codon bias, higher mF, or both (Sharp *et al*. 1986; Tuller *et al*. 2011; Zur and Tuller 2012). We had interpreted those results to be the consequence of long-term selection acting directly on codon bias and mF of each gene based on expression levels. Because our results are based on variation among alleles for each gene, we expected the effects of codon bias and mF on logPPR to be uniform across expression levels of genes. Instead, we see that the effects of codon bias and mF vary across expression levels of genes. To explain this result, we conjecture that at least 1 property of genes, which is correlated with expression levels (perhaps as a result of selection), mechanistically modifies how codon bias and mF are associated with protein accumulation. The phenotype of this property in highly expressed genes is to increase the impact of codon bias and mF on protein accumulation, and the phenotype of the property in low expression genes is to decrease the impact of codon bias and mF. We do not know what this property might be and suggest that it deserves further inquiry.

Our findings on codon bias agree with previous studies on how codon bias alone acts on protein expression. We found that tAI, which is solely based on tRNA supply estimated from tRNA gene copy numbers, had the most significant association with logPPR (Fig. 2 and Supplementary Fig. 2). Other measures of codon bias (CAI, nlCAI, and ntAI), which incorporate genomic usage of codons, were also significantly associated with logPPR, though to a lesser extent than tAI. The lower predictive ability of CAI, nlCAI, and ntAI is perhaps the consequence of codon usage being subject to multiple forces (e.g. selection for base composition (Lynch *et al*. 2008) or mRNA abundance (Cohen *et al*. 2018; Hia *et al*. 2019)). The higher predictive ability of tAI suggests that bias for codons with abundant cognate tRNAs is specifically and mechanistically related to protein accumulation.

Our refined understanding of the mechanisms by which codon bias acts alone on protein expression is in sharp contrast with our speculative understanding of how mF has the counterintuitive relationship of more stable structures associated with higher protein production (Zur and Tuller 2012). We are aware of 3 possible mechanistic models to explain this counterintuitive association: RNA homodimerization/aggregation avoidance, ribosome recycling via RNA circularization, and RNA structure refolding avoidance (see *Introduction*). Evidence exists that each could play a role; however, systematic evidence is lacking.

Our result that polymorphic mF is indeed positively associated with logPPR (Fig. 2b) was an important confirmation of the relationship of mF alone with protein levels. However, our examination of the interaction between codon bias and mF reframes the question about the mechanism of mF. We found that codon bias and mF act synergistically in their positive association with logPPR, that codon bias has no significant independent effects, and that the independent effects of mF are negative (positive slope for ensemble Δ*G* vs logPPR) but only weakly significant and inconsistent between logPPR and sqrtPPR models (Figs. 3 and 4 and Supplementary Figs. 3 and 4). Thus, the question of the mechanism of mF is more specifically a question about the mechanism by which codon bias and mF synergistically act to promote protein accumulation. This question remains unresolved. We have tRNA supply as an explanation for codon bias alone being positively associated with protein production, and we have several possible models for mF being positively associated with protein production. However, we lack mechanistic models that explain the strong synergy between codon bias and mF—strong enough that codon bias and mF have little to no independent effects.

It is noteworthy that the RNA structure refolding avoidance model described by Mao *et al*. (2014) is the only model we are aware of that explicitly predicts an interaction between codon bias and mF. Their simulations concluded that codon bias is expected to have a larger effect on protein synthesis rates when mF is high, but unlike our models, theirs do not predict that mF has larger effects when codon bias is high. Specifically, they predict that codon bias becomes the most important factor in determining protein accumulation when mF is large enough to result in a high density of ribosomes on the transcript; this high density prevents RNA secondary structure from reforming between adjacent ribosomes, keeping the pace of their translation high. Furthermore, they predict that mF at the 3′ end of transcripts would result in the biggest interaction between codon bias and mF. Although we did observe that codon bias has a larger effect when mF is high (Fig. 3a and Supplementary Fig. 3a), our results differed from Mao *et al*. (2014) in that we found that mF has a larger effect when codon bias is high (Fig. 3b and Supplementary Fig. 3b), that the interaction between codon bias and mF is bidirectional (Fig. 3c and Supplementary Fig. 3c), and that the regional effect of mF is highest in the CDS, not in the 3′ end of the transcript (Fig. 4c and Supplementary Fig. 4c). Our findings suggest that either codon bias or mF could play the role of the rate-limiting factor on protein accumulation. They also imply additional complexity in the role mF plays across the transcript than what was assumed in Mao *et al*.’s (2014) simulations. Our study will hopefully motivate future work in this area.

## Supplementary Material

iyad113_Supplementary_Data

## Data Availability

Data files, analysis scripts, and model details are available at https://github.com/anastacia9/bias_mF. Supplemental material available at GENETICS online.
