# Supplementary material for: Natural variation in codon bias and mRNA folding strength interact synergistically to modify protein expression in Saccharomyces cerevisiae
Source: Genetics. 2023 Jun 13;224(4):iyad113. doi: 10.1093/genetics/iyad113 (PMC10411576; doi:10.1093/genetics/iyad113)
Supplement: iyad113_Supplementary_Data [file iyad113_supplementary_data.zip › Figure_S1_GENETICS-2023-306086.pdf]

A

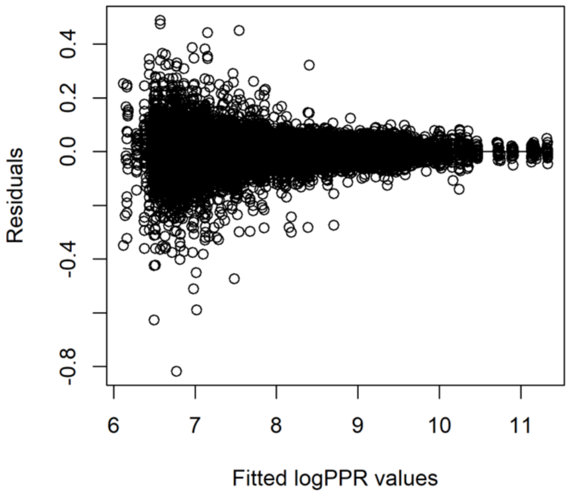

B

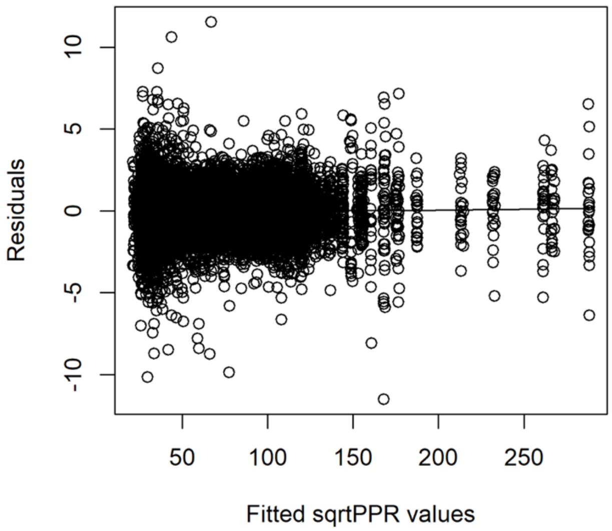

Figure S1. Log of protein per RNA (logPPR) model exhibits heteroskedasticity while square root of protein per RNA (sqrtPPR) model exhibits homoskedasticity. A, logPPR vs. residuals for CAI vs. logPPR linear mixed effects model. **B,** sqrtPPR vs. residuals for CAI vs. sqrtPPR linear mixed effects model.
