# Supplementary material for: Natural variation in codon bias and mRNA folding strength interact synergistically to modify protein expression in Saccharomyces cerevisiae
Source: Genetics. 2023 Jun 13;224(4):iyad113. doi: 10.1093/genetics/iyad113 (PMC10411576; doi:10.1093/genetics/iyad113)
Supplement: iyad113_Supplementary_Data [file iyad113_supplementary_data.zip › Figure_S2_GENETICS-2023-306086.pdf]

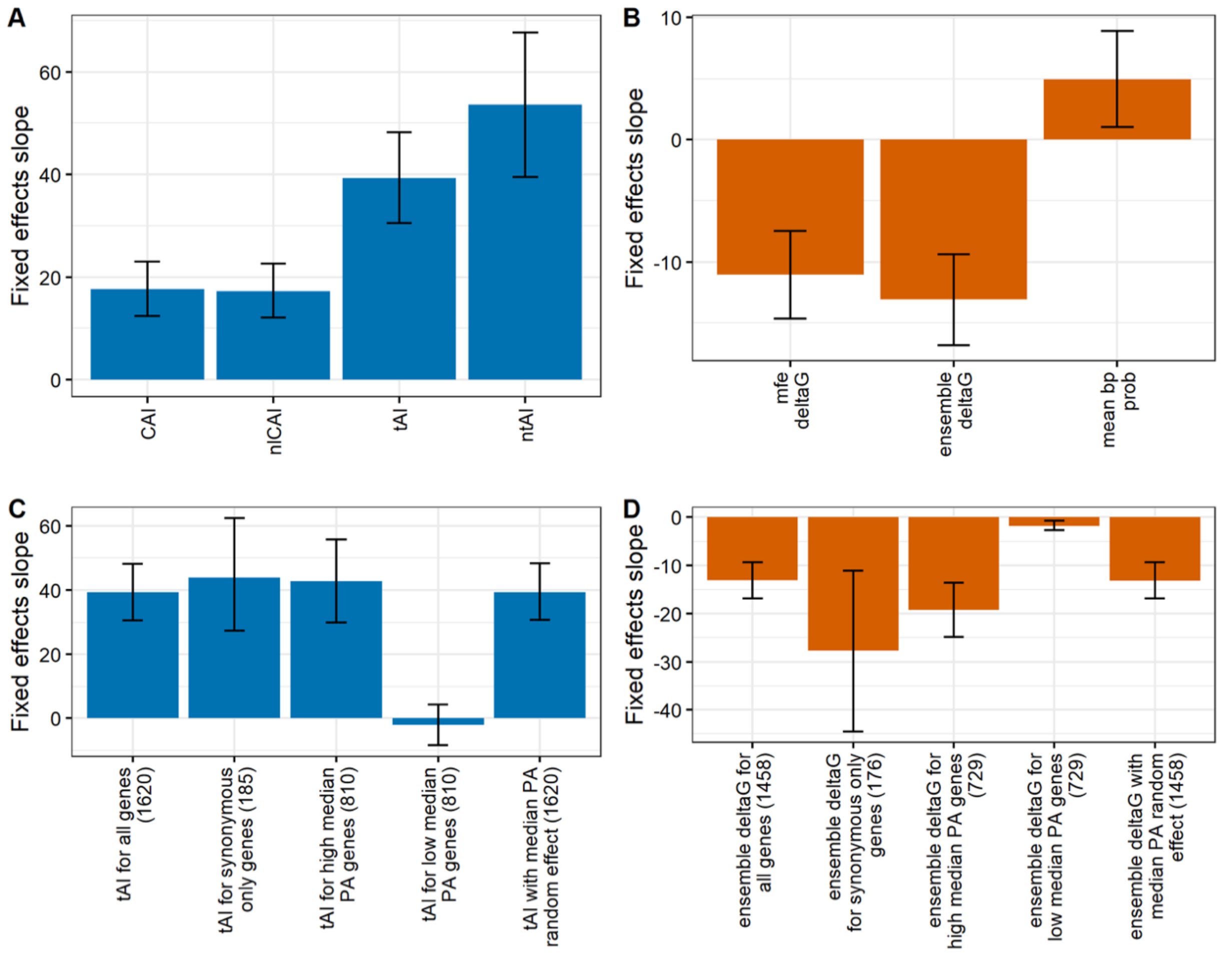

Figure S2. Polymorphic codon bias and mRNA secondary structure stability (mF) are each robustly associated with the square root of protein molecules per mRNA molecule (sqrtPPR). A, Fixed effects slope of each codon bias measure (codon adaption index (CAI), length normalized codon adaptation index (nlCAI), tRNA adaptation index (tAI), normalized tRNA adaptation index (ntAI)) is shown as the predictor of sqrtPPR in a linear mixed effects regression model. Models were computed using 1620 genes, where gene has a random effects slope and intercept. **B**, Fixed effects slope of each mF measure (minimum free energy ΔG (mfe ΔG), ensemble ΔG, and mean base-pair probability) as the predictor of sqrtPPR. Models were computed using 1458 genes, where gene has a random effects slope and intercept. **C**, Fixed effects slope of tAI as the predictor of sqrtPPR, where gene has a random effects slope and intercept. Models were run with all 1620 genes, 185 genes with synonymous and no non-synonymous polymorphisms, 810 genes with the highest across-isolate median protein abundance, 810 genes with the lowest across-isolate median protein abundance, and for all 1620 genes with additional random effects from median protein abundance. **D**, Fixed effects slope of ensemble ΔG as the predictor of sqrtPPR, where gene has a random effects slope and intercept.

Models were run with all 1458 genes, 176 genes with synonymous and no non-synonymous polymorphisms, 729 genes with the highest across-isolate median protein abundance, 729 genes with the lowest across-isolate median protein abundance, and for all 1458 genes with additional random effects from median protein abundance. Error bars represent 95% confidence intervals.
