# Supplementary material for: Natural variation in codon bias and mRNA folding strength interact synergistically to modify protein expression in Saccharomyces cerevisiae
Source: Genetics. 2023 Jun 13;224(4):iyad113. doi: 10.1093/genetics/iyad113 (PMC10411576; doi:10.1093/genetics/iyad113)
Supplement: iyad113_Supplementary_Data [file iyad113_supplementary_data.zip › Figure_S3_GENETICS-2023-306086.pdf]

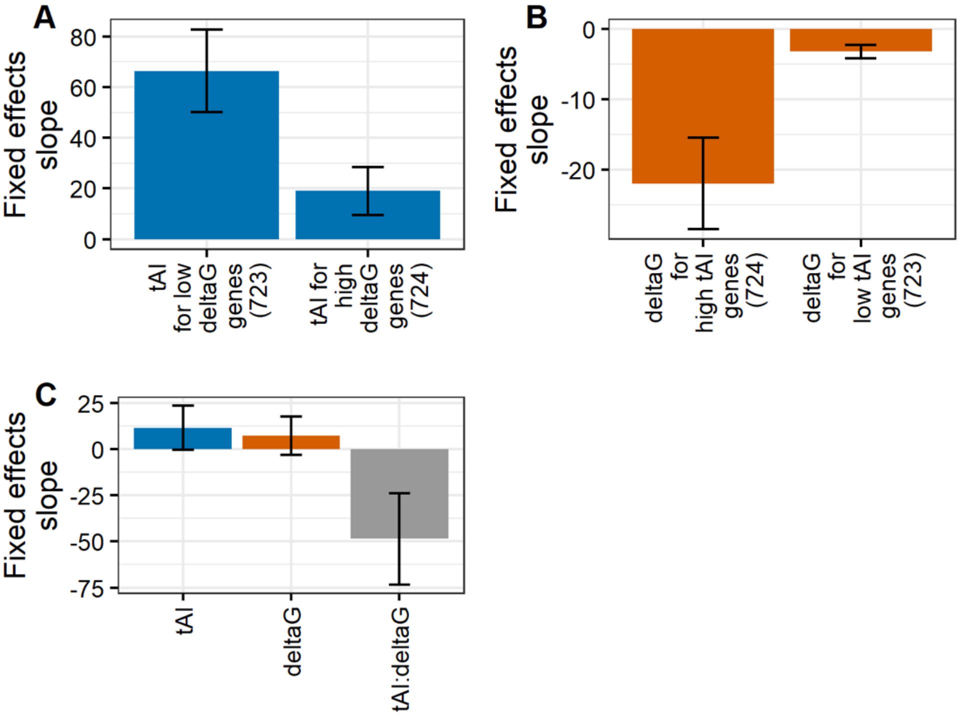

Figure S3. Polymorphic codon bias and mRNA secondary structure stability (mF) interact in association with protein expression as measured by the square root of protein molecules per mRNA molecule (sqrtPPR). A, Fixed effects slope of codon bias tRNA adaptation index (tAI) as the predictor of sqrtPPR in a linear mixed effects regression model for the bottom and top half of genes split by median (across alleles) mF ensemble ΔG. **B**, Fixed effects slope of ensemble ΔG as the predictor of sqrtPPR in a linear mixed effects regression model for the bottom and top half of genes split by median (across alleles) tAI. **C**, Fixed effects slope of tAI, ensemble ΔG, and tAI:ensemble ΔG interaction as the predictors of sqrtPPR in a linear mixed effects regression model. Error bars represent 95% confidence intervals.
