# Supplementary material for: Natural variation in codon bias and mRNA folding strength interact synergistically to modify protein expression in Saccharomyces cerevisiae
Source: Genetics. 2023 Jun 13;224(4):iyad113. doi: 10.1093/genetics/iyad113 (PMC10411576; doi:10.1093/genetics/iyad113)
Supplement: iyad113_Supplementary_Data [file iyad113_supplementary_data.zip › Figure_S4_GENETICS-2023-306086.pdf]

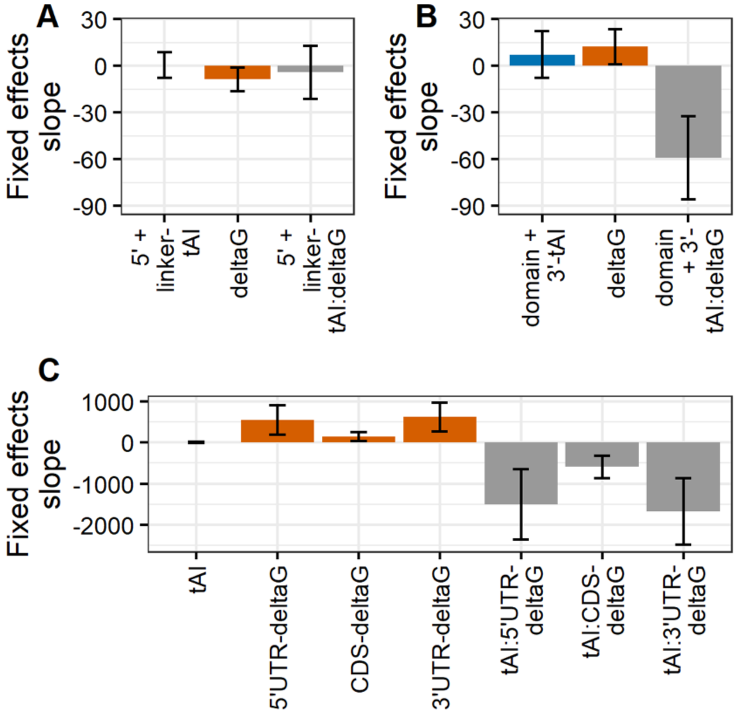

Figure S4. The effects of codon bias on square root protein molecules per mRNA molecule (sqrtPPR) are largely due to polymorphisms localized to domain encoding and 3' coding regions while the effects of mRNA folding stability (mF) on sqrtPPR are strongest in the CDS. To determine the localized effects of codon bias, we split coding sequences up into two regions: 5' coding (AUG up to first domain) plus linker (sequences between domains) and domain plus 3' coding (past last domain to stop codon). **A**, Fixed effects slope of 5' coding plus linker region codon bias tAI, whole transcript mF ensemble ΔG, and 5' coding plus linker region tAI:whole transcript ensemble ΔG interaction as predictors of square root protein molecules per RNA molecule (sqrtPPR) in a linear mixed effects regression model. **B**, Fixed effects slope of domain plus 3' coding region tAI, whole transcript ensemble ΔG, and domain plus 3' coding region tAI:whole transcript ensemble ΔG interaction as predictors of sqrtPPR in a linear mixed effects regression model. To determine the localized effects of mF, we calculated proportional sum of minimum free energy (psmfe) ΔG values for substructures spanning the 5' UTR, CDS, and 3' UTR. **C**, Fixed effects slope of CDS tAI, 5' UTR, CDS, and 3' UTR mF psmfe ΔG, and CDS tAI:5' UTR, CDS, and 3' UTR psmfe ΔG as predictors of sqrtPPR in a linear mixed effects regression model. Error bars represent 95% confidence intervals.
