# Supplementary material for: Natural variation in codon bias and mRNA folding strength interact synergistically to modify protein expression in Saccharomyces cerevisiae
Source: Genetics. 2023 Jun 13;224(4):iyad113. doi: 10.1093/genetics/iyad113 (PMC10411576; doi:10.1093/genetics/iyad113)
Supplement: iyad113_Supplementary_Data [file iyad113_supplementary_data.zip › Figure_S5_GENETICS-2023-306086.pdf]

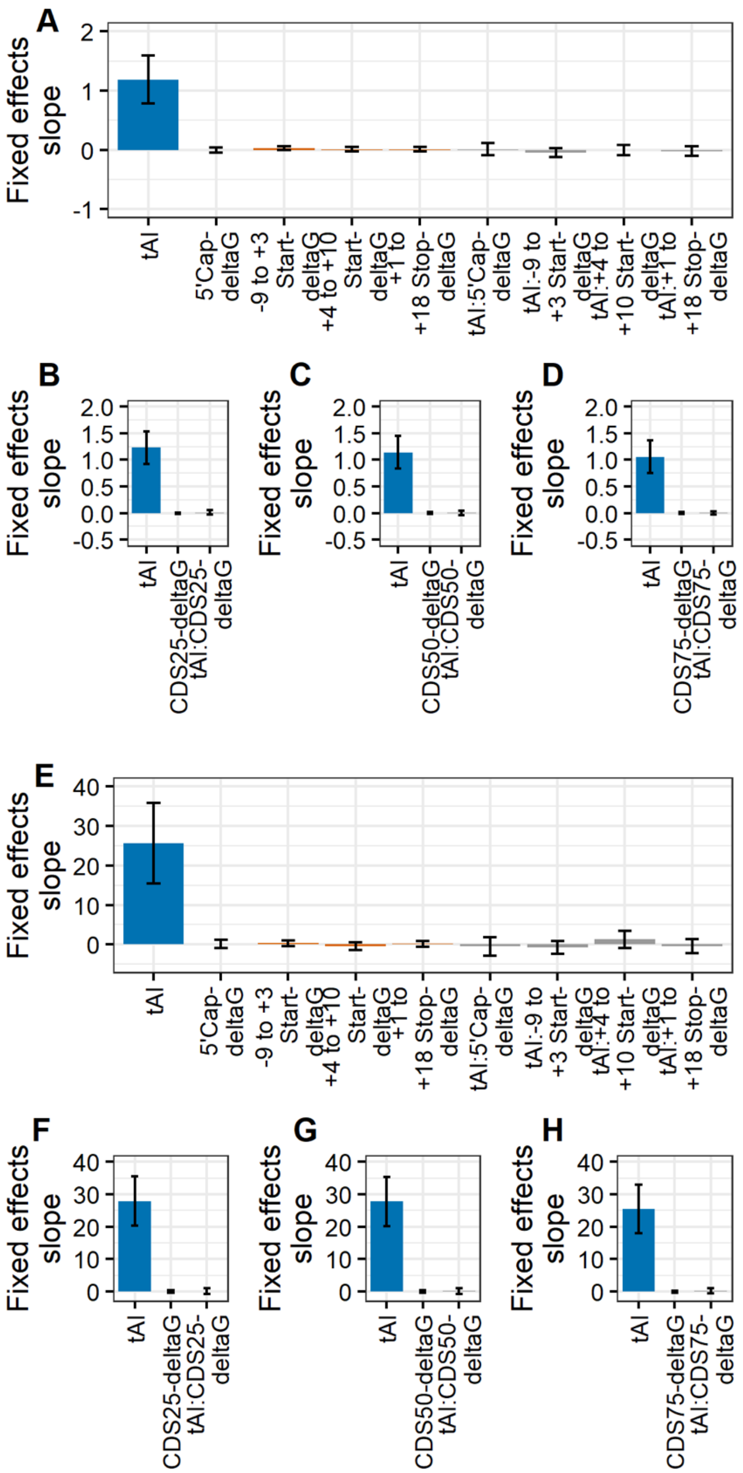

Figure S5. No evidence of fine-scale effects of mRNA folding stability (mF) on protein synthesis. To determine the fine-scale localized effects of mF, we calculated proportional sum of minimum free energy (psmfe) ΔG values for substructures spanning the 5’ cap (+1 to +10), just before and including the start codon (-9 to +3), just after the start codon (+4 to +10), and

just after and including the stop codon (+1 to +18). **A & E**, Fixed effects slope of CDS tAI, 5' cap, -9 to +3 start, +4 to +10 start, and +1 to +10 stop mF psmfe ΔG, and CDS tAI:5' cap, -9 to +3 start, +4 to +10 start, and +1 to +10 stop mF psmfe ΔG as predictors of logPPR (A) or sqrtPPR (E) in a linear mixed effects regression model. To evaluate our power to detect small-scale effects we sampled 40 bp regions located at 25%, 50%, and 75% of the total CDS length and calculated psmfe ΔG values for these regions. Fixed effects slope of CDS tAI, 25% (**B & F**), 50% (**C & G**), and 75% (**D & H**) CDS mF psmfe ΔG, and CDS tAI:25%, 50%, and 75% CDS mF psmfe ΔG as predictors of logPPR (B-D) or sqrtPPR (F-H) in a linear mixed effects regression model. Error bars represent 95% confidence intervals.
