# Supplementary material for: Natural variation in codon bias and mRNA folding strength interact synergistically to modify protein expression in Saccharomyces cerevisiae
Source: Genetics. 2023 Jun 13;224(4):iyad113. doi: 10.1093/genetics/iyad113 (PMC10411576; doi:10.1093/genetics/iyad113)
Supplement: iyad113_Supplementary_Data [file iyad113_supplementary_data.zip › Figure_S6_GENETICS-2023-306086.pdf]

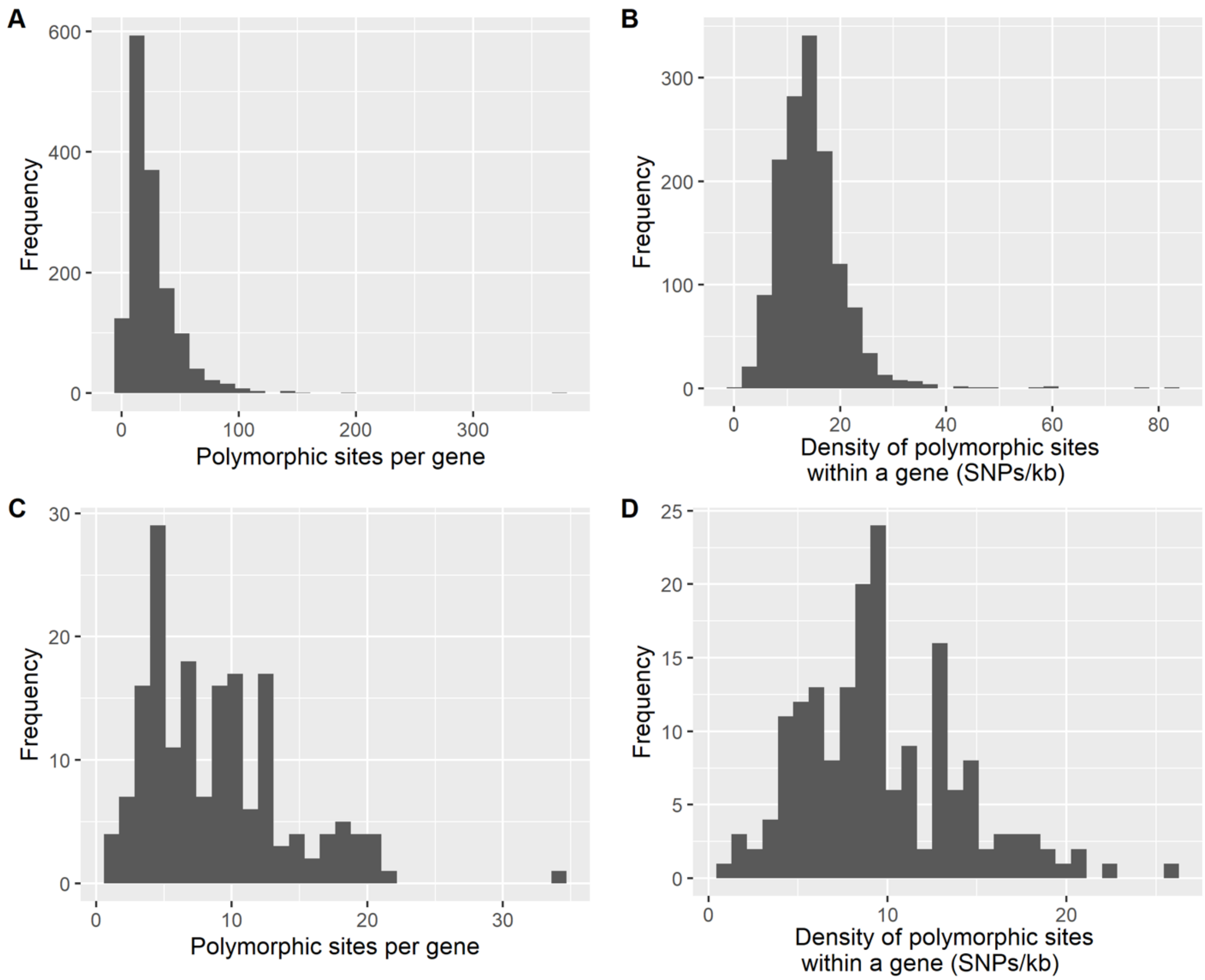

Figure S6. Distribution of SNP counts across genes. A, Histogram of the number of polymorphic sites per gene in the full set of 1458 genes with full length transcripts and at least one polymorphism. **B,** Histogram of the density (sites per kb) of polymorphic sites per gene in the full set of 1458 genes with full length transcripts and at least one polymorphism. **C,** Histogram of the number of polymorphic sites per gene in 176 synonymous only genes with full length transcripts and at least one polymorphism. **D,** Histogram of the density (sites per kb) of polymorphic sites per gene in 176 synonymous only genes with full length transcripts and at least one polymorphism.
